# Effect of the multiple sutures and suture-less wound closure on the wound healing after surgical extraction of the mandibular third molars: a systematic review and metaanalysis

**DOI:** 10.3389/froh.2026.1842421

**Published:** 2026-06-24

**Authors:** Somya Pande, Uday Londhe, Kalyani Bhate, Unnati Mehta, Aditya Jape

**Affiliations:** Regenerative Medicine Laboratory, Dr. D. Y. Patil Dental College and Hospital, Pune, India

**Keywords:** mandibular third molar surgery, meta-analysis, multiple sutures, postoperative pain, suture-less closure, swelling, systematic review, trismus

## Abstract

**Background:**

Surgical extraction of mandibular third molars is a common procedure performed in clinical practice yet it commonly yields postoperative pain, facial swelling, and trismus. While multiple-suture primary closure has traditionally been used, suture-less wound closure has emerged as an alternative.

**Aim:**

This systematic review and meta-analysis aimed to compare the effect of suture-less vs. multiple-suture wound closure on early postoperative pain, facial swelling, and trismus after mandibular third molar surgery.

**Material and methods:**

A comprehensive literature search was conducted in several databases for articles from January 2000 to December 2025. Only randomized controlled trials comparing suture-less and multiple-suture closure after surgical extraction of mandibular third molars were included. Meta-analyses were performed in RevMan 5.4 using a random-effects model due to substantial heterogeneity (*I*^2^ > 50%). Rob 2 tool was used for quality assessment of the included studies.

**Results:**

Ten RCTs were included. Meta-analysis showed that pain at 48 h was lower in the suture-less group (mean difference: −0.46; 95% CI: −3.69, 2.77; *p* = 0.33), but the difference was not statistically significant. Similarly, facial swelling at 48 h was lower in the suture-less group (mean difference: −0.56; 95% CI: −1.35, 0.22; *p* = 0.159), and trismus at 7 days was lower (mean difference: −0.38; 95% CI: −1.78, 1.22; *p* = 0.09), though neither reached conventional significance. Narrative synthesis indicated that suture-less closure was associated with comparable wound-healing and infection-related outcomes compared with multiple-suture closure.

**Conclusions:**

Suture-less techniques may be considered a safe and reasonable alternative as both approaches were associated with comparable early postoperative outcomes to multiple-suture primary closure in terms of pain, facial swelling, and trismus. Suture-less wound closure after surgical extraction of mandibular third molars can be a valid choice particularly when the goal is to minimize early inflammatory sequelae and patient discomfort.

**Systematic Review Registration:**

PROSPERO CRD42024563350.

## Introduction

One of the most common surgical procedures undertaken in the oral and maxillofacial practice is the surgical extraction of impacted mandibular third molars. However, postoperative morbidity which is mostly characterized by pain, facial edema and trismus is also a major problem that negatively affects patient comfort and quality of life. These sequelae can be primarily attributed to the degree of surgical trauma; manipulation of tissues and method of wound healing used after extraction (1).

Conventionally, primary closure with the use of sutures has been suggested to approximate the flap edges to achieve primary closure for complete or near-complete coverage of the socket, clot stabilization and to support healing through primary intention. However, the oral cavity has unique properties namely, continuous salivary flow, polymicrobial load, active functional movements, which can change the dynamics of wound healing compared to other surgical locations. Sutures might be associated with localized ischemia, drainage obstruction, and natural response amplification especially when used in tension. In addition, suture materials may serve as plaque-retentive niches, consequently enhancing the intensity of local inflammation and increasing the risk of developing postoperative complications like infection and alveolar osteitis (2).

Despite these factors, the sutureless or secondary closure methods in which flap repositioned without sutures or with only minimal stabilization have received more scholarly interest as an acceptable alternative approach. The technique allows the passive drainage of inflammatory exudate, decreases tissue tension, and can increase postoperative comfort. Several clinical studies and comparative studies have been done on the effectiveness of sutureless closure in reducing the postoperative morbidity; however, the data that have been obtained are not consistent. Although some studies indicate the decreasing pain, swelling, and trismus with the use of sutureless methods other researchers show that there is no statistically significant difference between the use of sutureless methods and traditional suturing techniques (3–8).

Recent systematic reviews have attempted to synthesize available evidence still there is variability in study designs, outcome assessment methods, and follow-up durations that has limited the ability to draw definitive conclusions (9). Furthermore, emerging modifications such as knotless sutures and tissue adhesives have added complexity to the decision-making process regarding optimal wound closure strategies (10–12).

On the basis of these inconsistencies and the ongoing debate regarding optimal wound closure, it is justified to provide an updated evaluation of the available evidence. Therefore, the present systematic review and meta-analysis aim to critically compare sutureless and multiple suture techniques following mandibular third molar surgery, with particular emphasis on postoperative pain, facial swelling, and trismus as key clinical outcomes.

## Material and methods

### Protocol

This systematic review followed the guidelines established by the Preferred Reporting Items for Systematic Reviews and Meta-Analyses (PRISMA) statement, ensuring a transparent reporting process. Additionally, the review protocol was registered on the PROSPERO platform, further enhancing transparency and accountability in the research. (Registration number: CRD42024563350).

### Focused question

“Is suture-less wound closure comparable to sutured wound closure following surgical extraction of the mandibular third molar?.” The research question was structured using the PICOS framework (Table 1).

**Table 1:** PICOS of the present study.

PICO		Description
Population	P	Patients requiring surgical extraction of mandibular third molars
Intervention	I	Sutureless wound closure
Comparison	C	Closure with suture
Outcome	O	Primary outcomes
		Post-operative healing outcomes: swelling, infection, trismus, wound dehiscence
		Secondary outcomes
		Bleeding
		Postoperative complications
Studies	S	Only randomized controlled trials

### Information sources and search strategy

We conducted a thorough search of prominent databases such as PubMed, Google Scholar, Scopus, Embase, EBSCO, Web of Science, Clinical Trials Registry India, and the Cochrane Central Register of Controlled Trials (CENTRAL. This search encompassed articles published between January 1, 2000, and December 31, 2025, and only articles published in English or those with detailed English summaries were considered. Additionally, references from both included and excluded studies were examined to identify any other relevant reports.

A set of keyword combinations; (“wisdom tooth”[Mesh] OR “third molar”[tiab] OR “mandibular third molar”[tiab]) AND (“tooth extraction”[Mesh] OR “surgical extraction”[tiab] OR “surgical removal”[tiab]) AND (“suture techniques”[Mesh] OR “sutureless”[tiab] OR “secondary intention healing”[tiab] OR “flap repositioned without sutures”[tiab] OR “sutureless wound closure”[tiab]) AND (“sutures”[Mesh] OR “sutured closure”[tiab] OR “primary closure”[tiab] OR “interrupted suture”[tiab] OR “resorbable suture”[tiab]) AND(“postoperative period”[Mesh] OR “postoperative complications”[Mesh] OR “facial swelling”[tiab] OR “trismus”[Mesh] OR “wound dehiscence”[Mesh] OR “infection”[Mesh] OR “alveolar osteitis”[Mesh]) were used to search the literature in all the databases to ensure that all relevant articles were screened.

### Eligibility criteria

Original, full-text, human studies published in English, in randomized controlled design were included. Studies comparing and evaluating the effectiveness of suture-free wound healing following the surgical extraction of the mandibular third molar were included. Systematic reviews, case reports, case series, or non-randomized trials, animal studies, articles not available in full-text format and publications in languages other than English were excluded. Studies that do not clearly classify the wound-closure technique as “suture-less” vs. “sutured” were excluded. Studies that do not report any of the predefined healing outcomes (swelling, infection, trismus, wound dehiscence) or provide only insufficient/unclear data were excluded. The inclusion and exclusion criteria were established based on the Study design, Participants, Interventions, Comparisons, and Outcomes (PICOS) framework. This structured approach ensures a comprehensive evaluation of relevant studies while maintaining clarity and focus on the objectives of the systematic review.

### Study selection

The selection process was carried out by two reviewers independently. The search process involved a systematic approach, organizing the articles into four distinct phases based on predetermined criteria. Initially, citations that did not meet the criteria during the first phase were immediately excluded. In the second phase, one reviewer meticulously screened the titles and abstracts of the retrieved articles to ensure they aligned with the established standards.

Articles that clearly failed to meet the criteria were swiftly discarded, while those requiring further consideration were closely reviewed. In cases of uncertainty, a second reviewer was consulted for further evaluation. In the third phase, two independent reviewers thoroughly assessed each article selected in the first phase to confirm compliance with the eligibility criteria. Articles with inappropriate study designs or insufficient baseline and endpoint outcome measures were excluded at this stage. Additionally, papers with inadequate referencing practices were consistently removed. Finally, the fourth phase involved a detailed review of the selected articles, focusing on extracting relevant data. The clinical procedures of each study were carefully analyzed, with particular attention to the interventions and outcomes investigated. If necessary, the third author was consulted for further input.

### Data collection process

Data collection was performed using a customized data extraction form. The first author carried out the first round of data extraction, after which the second author reviewed and improved it. For every full-text article that satisfied the predetermined inclusion criteria, data extraction was done. The information gathered included key details such as study characteristics (author names, publication year, and country), participant information or sample population (sample size, age), intervention method, comparator, outcome evaluation, results, and the study conclusions.

### Risk of bias and quality of assessment

To determine the risk of bias for included randomized controlled trials, a revised Cochrane RoB 2 tool was used. RoB 2 evaluates bias across five domains. Each domain was judged as “low risk of bias,” “some concerns,” or “high risk of bias,” based on the signalling questions and algorithms provided in the RoB 2 guidance (13).

### Data synthesis

The data from the selected studies were combined using a narrative approach, focusing on the types of interventions, participant characteristics, and clinical outcomes. Focus on key elements such as intervention details, participant characteristics (age, gender), inclusion and exclusion criteria, study designs, outcomes, and the overall effects of the interventions.

### Statistical analysis

The meta-analyses, using random effects model, were applied with RevMan 5.4 (RevMan 5.4, The Nordic Cochrane Centre, Copenhagen). Heterogeneity was assessed by *Q* test and quantified with *I*^2^ statistics for analyses, if the test showed substantial heterogeneity (*I*^2^ > 50%), a random effects model was applied, or else (*I*^2^ ≤ 50%), a fixed effects model would be used. Pain, facial swelling and trismus among the subjects treated with sutureless and multiple suture groups were considered as the main outcome.

### Certainty assessment

The GRADE (Grading of Recommendations, Assessment, Development and Evaluation) approach is used to rate the certainty of evidence for each key outcome.

## Result

### Literature search

**Figure 1:**
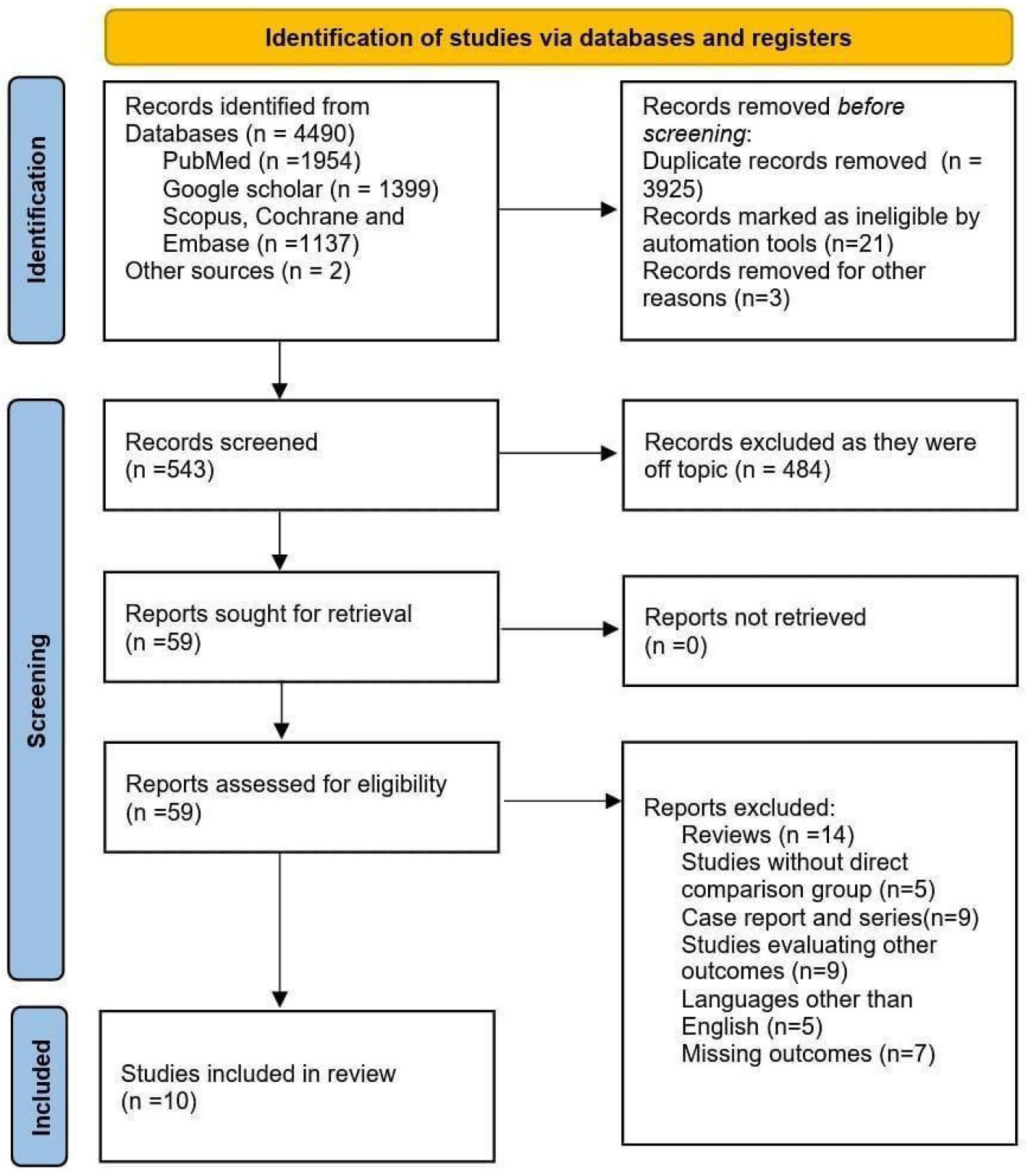
Summary of literature search.

The kappa value was 0.98; therefore, an agreement amongst the 3 investigators was acceptable. Through electronic searches and other sources, 4492 articles were identified. After removing duplicates, 543 articles remained. The titles and abstracts of the 543 records were examined based on predefined eligibility criteria. As a result, 484 articles were excluded because they were off-topic. The full text of the remaining 59 articles was carefully read by two reviewers for potential inclusion. The articles were narrowed down to 10. Thus, 10 studies were selected to draw the results of the systematic review. The process of study selection is documented in the PRISMA flowchart in Figure 1.

### General characteristics of the included studies

A total of ten studies were included. The characteristics of the included studies are summarised in Tables 2, 3. All 10 included studies are randomized controlled trials (RCTs) comparing suture-less wound closure vs. sutured closure after surgical extraction of mandibular third molars. The total combined sample across studies is 556 patients/extraction sites, with individual study sizes ranging from 30 to 94 patients. Overall, suture-less closure tends to either reduce or not worsen early pain compared with multiple-suture closure, but effects are not uniform. The majority of study authors conclude that suture-less or single-suture techniques are at least non-inferior and often superior in reducing early postoperative pain, swelling, and trismus, while maintaining acceptable or improved wound healing.

**Table 2:** General characteristics of included studies.

Sno	Author, year and country	Study design	Study population	Study groups	Sample characteristics
1	Katta et al. (14) 2018	RCT	Total sample -30 patients	Group A: suture, (*n* = 15) Group B: suture-less (*n* = 15)	Age—18 to 44 years; Mean age—25.2 years.
	India				
2	Takadoum et al. (3) 2022	RCT	Total sample -94 patients	Group A: suture, (*n* = 44) Group B: suture-less (*n* = 50)	Age—<18 years’ old; mean age -17 (15–19); Females- Suture group-59.6% Sutureless group-73.1%
	France				
3	Mahat et al. (15) 2020	RCT	Total sample -48 patients	Group A: suture, (*n* = 24) Group B: suture-less (*n* = 24)	Mean age- Suture group -28.63 ± 8.75 Sutureless -28.38 ± 6.79; Females- Suture group -8 Sutureless group -12
	Nepal				
4	Osunde et al. (11) 2011	RCT	Total sample -50 patients	Group A: suture, (*n* = 25) Group B: suture-less (*n* = 25)	Mean age- Suture group -26 ± 4.75 Sutureless -25.8 ± 4.28; Females- Suture group -12 Sutureless group -11
	Nigeria				
5	Rodrigues et al. (4) 2023	RCT	Total sample -31 patients	Group A: suture, (*n* = 31) extraction site Group B: suture-less (*n* = 31) extraction site	Mean age- 22.04 ± 2.45: Females-12 Males-19
	Italy				
6	Chukwuma et al. (6) 2022	RCT	Total sample -74 patients	Group A: suture, (*n* = 37) Group B: suture-less (*n* = 37) e	Mean age- 30.2(±8.3) years: Females-35 Males-39
	Nigeria				
7	Osunde, et al, (17) 2012	RCT	Total sample -80 patients	Group A: suture, (*n* = 40) Group B: suture-less (*n* = 49)	Mean age- 27.9 (5.47) years: Females-38 Males-42
	Nigeria				
8	Alkadi et al. (8) 2019	RCT	Total sample -35 patients	Group A: suture, (*n* = 35) extraction site Group B: suture-less (*n* = 35) extraction site	Mean age- 26.6 (SD = 4.85) years: Females-25 Males-10
	Ireland				
9	Gay-Escoda et al, (7) 2015	RCT	Total sample -40 patients	Group A: suture, (*n* = 40) Group B: suture-less (*n* = 40)	Mean age- 25.2 (SD = 4.85) years: Females-21 Males-19
	Spain				
10	Hashemi et al. (5) 2012	RCT	Total sample -30 patients	Group A: suture, (*n* = 30) extraction sites Group B: suture-less (*n* = 30) extraction sites	Mean age- 22 years: Females-9 Males-22
	Iran				

**Table 3:** Outcomes assessed in the included studies.

Sno	Author, year and country	Pain intensity	Trismus	Facial edema	soft tissue healing	Conclusion
1	Katta et al. (14) 2018	Pain was greater in suture closure than sutureless at 48 h and 7 days but was not statistically significant.	Trismus was greater in suture closure than sutureless at 48 h and 7 days but was not statistically significant	No differences in facial swelling between the sutured and non-sutured sides at 48 h and at 7 days (*p* > 0.05).	soft tissue healing was excellent in sutureless group as compared to control group after one month.	Sutureless technique is superior in decreasing postoperativ e pain and enhancing wound healing.
	India					
2	Takadoum et al. (3) 2022	pain were not different between groups on day 0, 3 and 31	The variation in trismus was not significant between the two groups on day 0, 3 and 31	No differences were seen for swelling among groups on day 0, 3 and 31	No differences were seen for healing among groups on day 0, 3 and 31	Sutureless removal of third molars is thus a reliable technique without negative consequence
	France					
3	Mahat et al. (15) 2020	mean NRS score was significantly higher in suture group on the 1st postoperative day	The mouth opening was significantly lesser in suture less group	The swelling was significantly more in the multiple sutures group		The use of sutureless technique after third molar surgery can minimize postoperativ e morbidity
	Nepal					
4	Osunde et al. (11) 2011	The pain score was higher in the suture group than sutureless group and was statistically significant on day 1 and 2.	The trismus was higher in the suture group than sutureless group and was statistically significant on day 1, 2, and 3.	The edema was higher in the suture group than sutureless group and was statistically significant on day 1, 2, and 3.	-	Single suture technique was better’ than sutureless
	Nigeria					
5	Rodrigues et al. (4) 2023	The pain score was higher in the suture group than sutureless group and was statistically significant on 24 h and 48 h	The trismus was higher in the suture group than sutureless group and was statistically significant on 24 h and 48 h	The edema was higher in the suture group than sutureless group and was statistically significant on 24 h and 48 h	Better healing noted on sutureless group	Single suture technique superior to the primary closure in reduction of post-operati ve pain, swelling and trismus.
	Italy					
6	Chukwuma et al. (6) 2022	The sutureless group had statistically significantly higher postoperativ e pain on days 1, 3, 4 and 5 (*P* < 0.05)	The sutureless group had lesser severity of trismus on day 7 (*P* < 0.05) than the complete closure group.	There was no significant difference in swelling	-	Compared with the complete closure group, the sutureless group had similar severity of swelling, less trismus but had higher pain severity in the week following M3 surgery.
	Nigeria					
7	Osunde, et al, (17) 2012	Postoperativ e pain was significantly less in the experimental group compared to the controls on post- operative days 1 and 2 (*p* < 0.05).	The sutureless group had lesser severity of trismus on 24 h and 48 h (*P* < 0.05) than the complete closure group.	The sutureless group had lesser edema on 24 h and 48 h (*P* < 0.05) than the complete closure group.	-.	There is less postoperativ e pain, swelling and trismus with the suture-less technique in third molar surgery.
	Nigeria					
8	Alkadi et al. (8) 2019	Pain severity was greater on the non-sutured side		No differences in edema between the sutured and non-sutured sides at all evaluation days	No differences in soft tissue healing between the sutured and non-sutured sides at all evaluation days	Suture-less technique is superior in reduction of post-operati ve pain, and improving wound healing during early post-operati ve period.
	Ireland					
9	Gay-Escoda et al, (7) 2015	No statistically significant differences were found to be related to pain (*p* < 0.06) at 48 h and 7 days between the groups	There were no significant differences for trismus between none of them by measuring the mouth opening (*p* < 0.71) before surgery, at 48 h and at 7 days after surgery,	There were no significant differences for horizontal (*p* < 0.73), verti- cal (*p* < 0.37) and oblique (*p* < 0.83) facial measuremen ts for swelling among groups		Partial closure of the flap without suturing the relieving incision after surgical extraction of lower third molars reduces operating time and it does not produce any postoperativ e complicatio ns compared with com- plete closure of the wound.
	Spain					
10	Hashemi et al. (5) 2012	No statistically significant differences were found to be related to pain (*p* < 0.05) at 3rd and 7th day among the groups	There were no significant differences for trismus between groups	There were no significant differences for swelling among groups	-	-
	Iran					

### Metaanalysis

**Figure 2:**
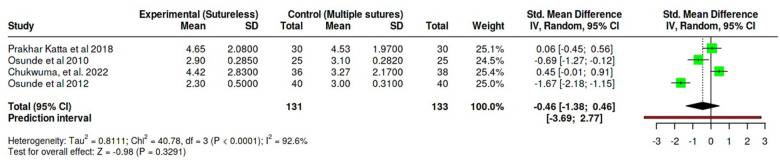
Forest plot depicting comparison of pain at 48 h among the subjects treated with sutureless and multiple suture groups.

**Figure 3:**
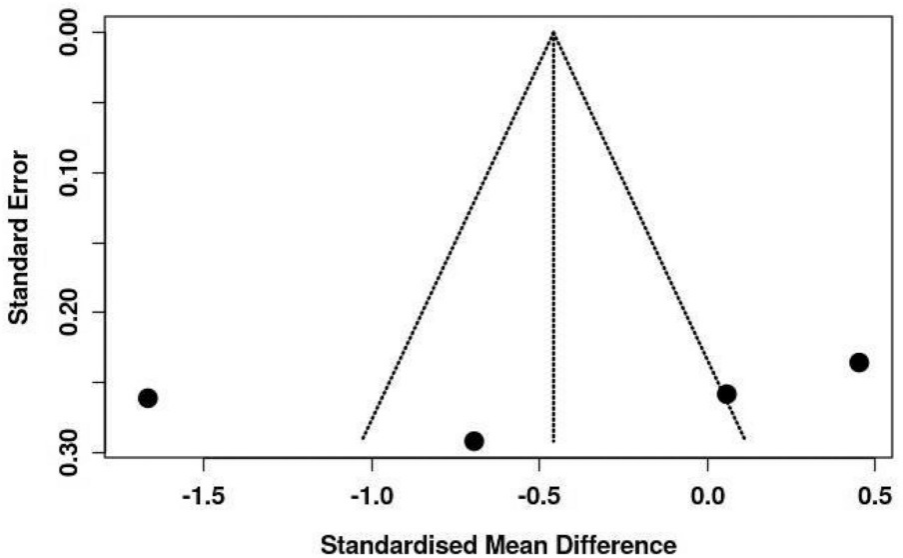
Funnel plot representing assessment of publication bias for pain at 48 h.

**Figure 4:**
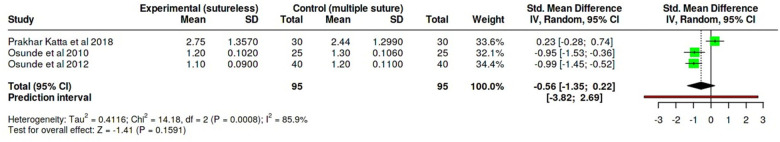
Forest plot representing comparison of facial swelling at 48 h among the subjects treated with sutureless and multiple suture groups.

**Figure 5:**
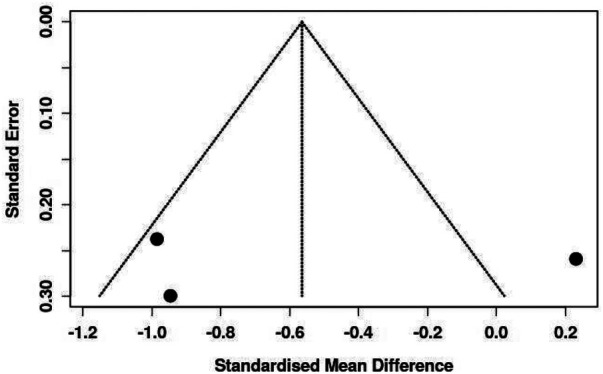
Funnel plot representing assessment of publication bias for facial swelling at 48 h.

**Figure 6:**
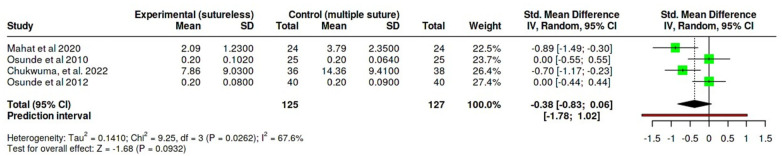
Forest plot representing comparison of trismus at 7th day among the subjects treated with sutureless and multiple suture groups.

**Figure 7:**
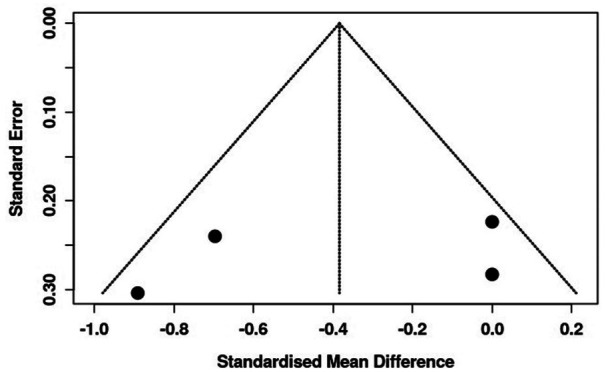
Funnel plot representing assessment of publication bias for trismus at 7th day.

1. **Comparison of pain at 48 h among the subjects treated with sutureless and multiple suture groups:** With the meta-analysis conducted for selected studies, heterogeneity was more than 50% (*I*^2^ = 92.8%); hence, a random effect model was applied. A total of 4 studies: 131 subjects in the Experimental (Sutureless) cohort and 133 subjects in the Control (Multiple sutures) cohort were included for metaanalysis. The pain at 48 h was lower in the sutureless group compared multiple suture group with a mean difference of −0.46 (95% CI = −3.69 to 2.77; *Z* value = −0.98); however, the difference between the two groups was statistically non-significant (*p* = 0.33) (Figure 2). The funnel plot to assess the publication bias for pain at 48 h showed less possibility of publication bias since the funnel plot was symmetric in shape. The Egger's test does not indicate the presence of funnel plot asymmetry (intercept: −22.91, 95% CI: −72.54 to 26.73, *t*: −0.905, *p*-value: 0.461) (Figure 3).
2. **Comparison of facial swelling at 48 h among the subjects treated with sutureless and multiple suture groups:** With the meta-analysis conducted for selected studies, heterogeneity was more than 50% (*I*^2^ = 85.9%); hence, a random effect model was applied. A total of 3 studies: 95 subjects in the Experimental (Sutureless) cohort and 95 subjects in the Control (Multiple sutures) cohort were included for metaanalysis. The facial swelling at 48 h was lower in the sutureless group compared multiple suture group with a mean difference of −0.56 (95% CI = −1.35 to 0.22; *Z* value = −1.41); however, the difference between the two groups was statistically non-significant (*p* = 0.159) (Figure 4). The funnel plot to assess the publication bias for facial swelling at 48 h showed less possibility of publication bias since the funnel plot was symmetric in shape. The Egger's test does not indicate the presence of funnel plot asymmetry (intercept: −0.38, 95% CI: −45.78 to 45.03, *t*: −0.016, *p*-value: 0.99) (Figure 5).
3. **Comparison of trismus at 7th day among the subjects treated with sutureless and multiple suture groups:** With the meta-analysis conducted for selected studies, heterogeneity was more than 50% (*I*^2^ = 67.6%); hence, a random effect model was applied. A total of 4 studies: 125 subjects in the Experimental (Sutureless) cohort and 127 subjects in the Control (Multiple sutures) cohort were included for metaanalysis. The trismus at 7th day was lower in the sutureless group compared multiple suture group with a mean difference of −0.38 (95% CI = −1.78 to 1.22; *Z* value = −1.68); however, the difference between the two groups was statistically non-significant (*p* = 0.09) (Figure 6). The funnel plot to assess the publication bias for trismus at 7th day showed less possibility of publication bias since the funnel plot was symmetric in shape. The Egger's test does not suggest the presence of funnel plot asymmetry (intercept: −4.96, 95% CI: −20.86 to 10.94, *t*: −0.611, *p*-value: 0.603) (Figure 7).

### Risk of bias

**Figure 8:**
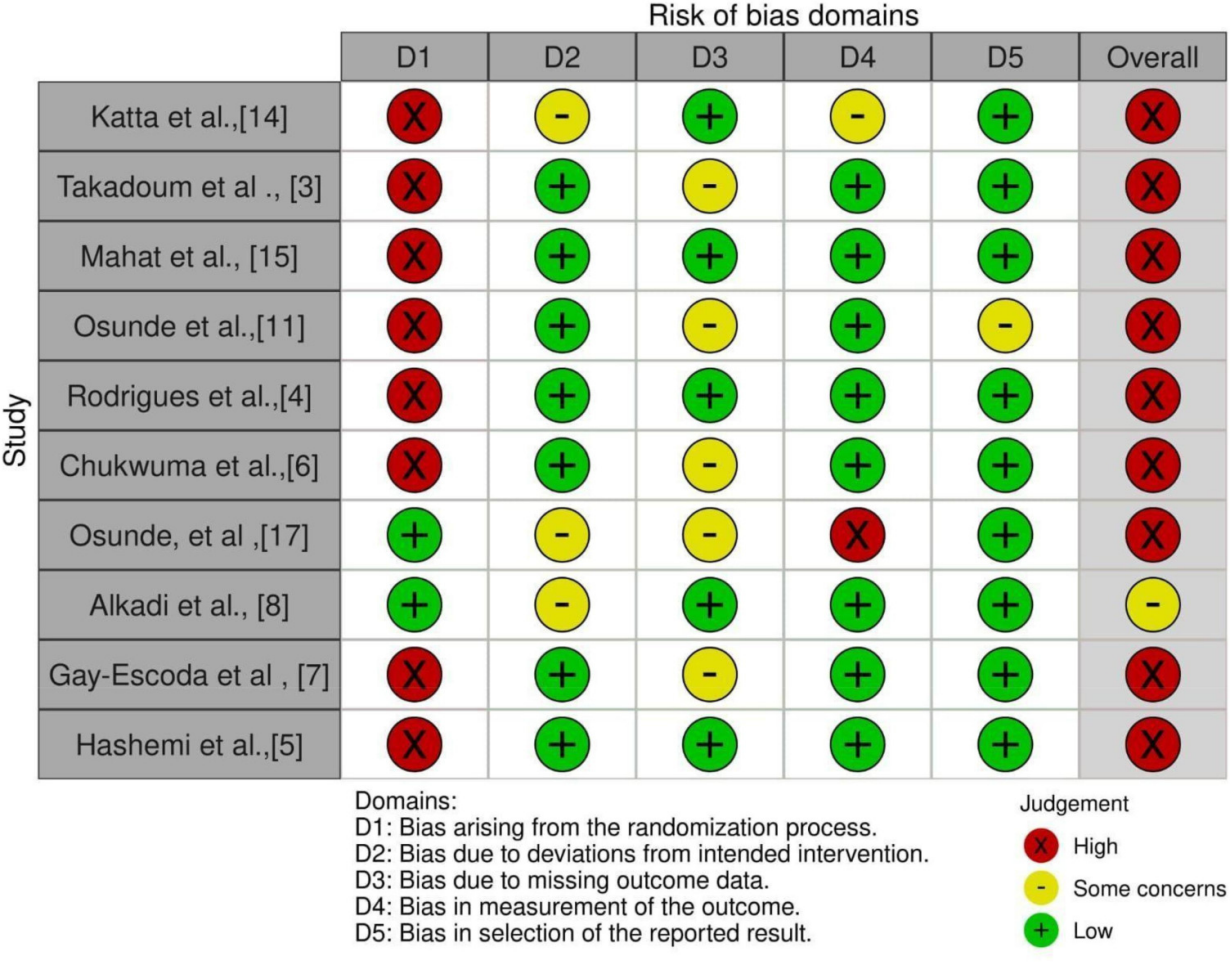
Risk of bias assessment of the included individual studies.

**Figure 9:**
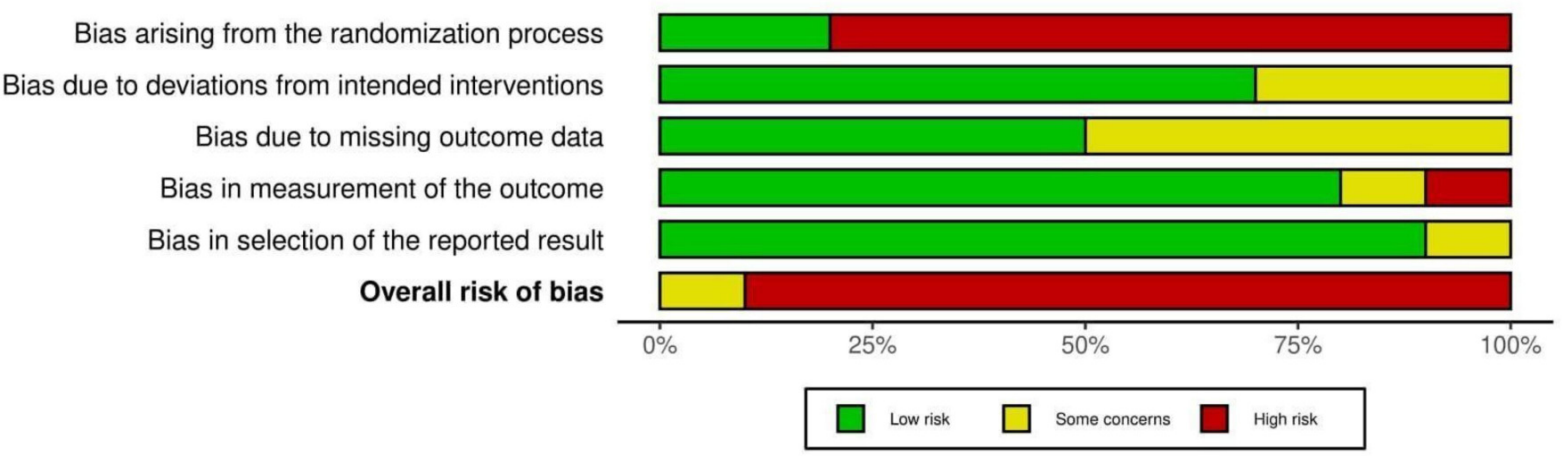
Summary plot of risk of bias assessment with Rob 2 tool.

The risk of bias in the 10 included randomized controlled trials was assessed using the Cochrane RoB 2 tool. Several studies were rated “High” in D1, suggesting possible problems in allocation sequence generation or allocation concealment. In contrast, domains related to deviations from interventions (D2) and missing data (D3) were often rated “Low” or “Some concerns,” indicating generally acceptable adherence to protocol and follow-up. Domain D4 (outcome measurement) showed variability, with some trials rated as “Low,” others as “Some concerns,” and a few as “High,” reflecting differences in blinding and standardization of pain, swelling, and trismus assessment. Finally, D5 (selection of reported results) was mostly judged as “Low” or “Some concerns,” with no clear evidence of widespread selective reporting of the main outcomes. Only Alkadi et al. received an overall rating of “Some concerns,” reflecting a more robust methodological profile across domains, while the remaining nine studies were classified as “High” risk due to at least one major flaw in randomization or outcome measurement. This pattern suggests that the pooled estimates (pain, swelling, trismus) should be interpreted with moderate to low certainty, acknowledging the influence of methodological limitations despite the consistency of direction of effect across studies (13) (Figure 8, 9).

### Certainty assessment

The certainty of evidence for each pooled outcome was evaluated using the Grading of Recommendations Assessment, Development and Evaluation (GRADE) approach.

1. **Risk of bias—Downgrade by 1 level (serious):** Although all 10 included studies were randomized controlled trials, the RoB 2.0 assessment revealed that 9 of the 10 studies were judged at “High” overall risk of bias, primarily due to unclear randomization procedures, allocation concealment, and lack of blinding of participants, surgeons, and outcome assessors, which is common in surgical-extraction trials. Only Alkadi et al. (2019) were rated as having “Some concerns” overall, with relatively better methodological quality.

Given that the majority of studies were not at uniformly low risk of bias across all domains, the certainty of evidence for each outcome was downgraded by one level for serious risk of bias.

2. **Inconsistency—Downgrade by 1 level (serious):** Considerable statistical heterogeneity was observed (Pain at 48 h: *I*^2^ = 92.8%, facial swelling at 48 h: *I*^2^ = 85.9%, and trismus at 7 days: *I*^2^ = 67.6%), indicating extreme variability in effect sizes across studies. Clinically relevant moderators (e.g., flap design, impaction difficulty, analgesic protocols, and time-points of assessment) were not consistently reported or prespecified, and subgroup analyses were limited by small numbers. This unexplained heterogeneity warranted downgrading the certainty of evidence by one level for serious inconsistency.
3. **Indirectness—No downgrade:** The population, intervention, comparator, and outcome were directly aligned with the review question. All included studies addressed the same clinical scenario and outcome definitions, and therefore no concerns regarding indirectness were identified. No downgrade was applied for this domain. Therefore, no concerns regarding indirectness were identified.
4. **Imprecision—No downgrade:** For all pooled continuous outcomes, the confidence intervals were wide and included both clinically meaningful benefit and no effect, indicating imprecision due to small sample sizes and low event rates in binary outcomes. Even though the effect direction generally favored suture-less closure, the magnitude of benefit remained uncertain. Thus, the certainty of evidence was downgraded by one level for serious imprecision.
5. **Publication bias—Not downgraded (undetected):** Formal assessment of publication bias using funnel plots and Egger's tests was conducted for each outcome. The funnel plots were visually symmetric, and Egger's tests were non-significant (*p* > 0.05) for pain, facial swelling, and trismus, indicating no strong evidence of publication bias.

**Overall certainty of evidence:** As the included evidence was derived from randomized controlled trials, the certainty of evidence was initially rated as high. However, the certainty were then adjusted as follows (Table 4):

- Pain at 48 h: downgraded by 1 level (serious risk of bias), 1 level (serious inconsistency), and 1 level (serious imprecision)- Low certainty.
- Facial swelling at 48 h: downgraded similarly- Low certainty.
- Trismus at 7 days: downgraded similarly- Low certainty.

**Table 4:** Assessment of certainty of evidence through GRADE analysis:.

Outcome	No. of studies (participants)	Study design	Risk of bias	Inconsistency	Indirectness	Imprecision	Publication bias	Pooled effect estimate (95% CI)	Certainty of evidence (GRADE)
Pain at 48 h	4 (264 patients)	Randomized controlled trials	Serious^†^	Serious^‡^	Not serious	Serious^‡^	Undetected	MD = −0.46 (−3.69, 2.77)	⊕⊕⊕◯ Low
Facial swelling at 48 h	3 (190 patients)	Randomized controlled trials	Serious^†^	Serious^‡^	Not serious	Serious^‡^	Undetected	MD = −0.56 (−1.35, 0.22)	⊕⊕⊕◯ Low
Trismus at 7 days	4 (252 patients)	Randomized controlled trials	Serious^†^	Serious^‡^	Not serious	Serious^‡^	Undetected	MD = −0.38 (−1.78, 1.22)	⊕⊕⊕◯ Low

† **Risk of bias:** Most included trials were judged to have some concerns according to the RoB 2.0 tool, primarily due to unclear allocation concealment and lack of blinding of participants and operators, which is inherent in surgical and laser-assisted orthodontic interventions.

‡ **Inconsistency:** Considerable heterogeneity was observed among studies (high *I*^2^ values), indicating substantial variability in effect sizes that could not be fully explained.

## Discussion

Ensuring that wound edges are properly closed and held securely in place is essential for achieving successful healing after any surgery. In dental practice, the surgical extraction of third molars is a routine procedure commonly performed in clinical settings (18). Pain, swelling, and decreased mouth opening, are inevitable consequences following this procedure postoperatively (19).

To allow healing of the extraction socket by primary intention, and to prevent exposure of denuded bone and exposed root surfaces caused by the surgical intervention, flap margins created by surgical incisions are preferably approximated and sutured. Suturing plays an important role in healing of wounds, enabling reconstruction and reassembly of tissue separated by a surgical procedure or a trauma, and at the same time facilitating and promoting healing and hemostasis (20).

However, Suturing in the oral cavity behaves differently from those used for other parts of the body due to differences in the quality of tissue involved, constant presence of saliva, high level of vascularisation and the presence of functions of speech, chewing and swallowing (21). Suturing knots act as an area of plaque-accumulation (22). Inadequate plaque removal can lead to local infection, inflammation, edema, clot necrosis, alveolar osteitis, and pain (22). Nonetheless, Suturing also creates tension which subsequently leads to restricted mouth opening. To avoid these, sutureless closure with minimal manipulation of soft tissues and ease of oral hygiene maintenance have been utilized in the recent decade (23, 24). Thereby we aimed to systematically evaluate the effects of sutureless and multiple sutures after the third molar surgeries with respect to pain, facial swelling and trismus. In this study, a total of 10 studies were included.

### Pain

In the present metaanalysis, the pain at 48 h was lower in the sutureless group compared to multiple suture groups. The pooled results did not show statistically significant differences between the groups. These findings are in accordance with the current research, Alkadi et al. (8) and Osunde et al.2011 (16) reported that the pain severity was greater on the sutureless group. Osunde et al. (16) in their randomized controlled study reported that the pain was significantly higher in the Suture group up to Day 2, with no further difference up to Day 7. Nonetheless, Mahat et al. (15) noted that the pain was significantly greater in the first postoperative day in the multiple sutures group yet the difference was not statistically significant on other postoperative days. Chukwuma et al. (6) study demonstrated that pain score was higher in sutureless groups on all postoperative days, and this difference was statistically significant only on postoperative days 1, 3, 4 and 5. However, the rate of decline in pain intensity was higher in sutureless groups as seen by the greater steepness of the downward slope indicating accelerated recovery dynamics possibly from unobstructed drainage.

In contrast to the present findings, studies by Katta et al. (14), and Takadoum et al. (3) revealed no statistically significant differences at 48 h but after 7 days the pain was statistically significant (*p* < 0.05). This reflects the delayed benefits of secondary closure in minimizing chronic inflammation and scar formation. These heterogeneities with respect to pain variables underscore the need for standardized pain scales (e.g., VAS) and timing in future trials, as methodological factors like patient demographics, impaction complexity, and analgesic protocols moderate outcomes in third molar surgery wound closure comparisons.

### Facial swelling

In the present metaanalysis, the facial swelling at 48 h was lower in the sutureless group compared to multiple suture groups. The pooled results did not show statistically significant differences between the groups. In contrast to the present findings, studies by Katta et al. (14), Alkadi et al. (8), and Takadoum et al. (3) revealed no differences in facial swelling between the sutured and non-sutured sides at 48 h and at 7 days. However, Mahat et al. (15) and Chukwuma et al. (6) noted that the swelling was significantly more in the multiple sutures group when measured from the gonion to lateral canthus on all postoperative days. This could be due to suture-induced tension compressing tissues, impairing venous return, and prolonging inflammatory peaks. The favorable results of the present study reflects reduced local inflammatory response and tissue manipulation associated with secondary intention healing, allowing better lymphatic drainage and less edema accumulation compared to primary closure with multiple sutures.

### Trismus

In the present metaanalysis, the trismus at 48 h was lower in the sutureless group compared to multiple suture groups. The findings of the meta-analysis stems from reduced masseter muscle inflammation and edema due to secondary closure, which minimizes tissue distortion and muscular tension compared to multiple sutures that can tether adjacent structures. In contrast to the present findings, studies by Katta et al. (14), and Takadoum et al. (3) revealed no significant differences in mouth opening at 48 h and at 7 days after surgery. Chukwuma et al. reported that the sutureless group had less trismus than the complete closure group postoperatively.

### Bleeding

Among the included studies, postoperative bleeding was only reported in few studies and was generally described as an infrequent or minor event, with no studies reporting a statistically significant difference between suture-less and sutured groups. Hashemi et al. (5), Alkadi et al. (8), Rodrigues et al. (4), and Osunde et al. (16) explicitly noted similar low rates in both suture-less and sutured extraction sites.

### Postoperative complications

Across the included randomized trials, the most frequently reported complications were: Alveolar osteitis (dry socket), Postoperative infection/local inflammation, Wound dehiscence/delayed healing and Transient sensory disturbances (lingual or inferior alveolar nerve–related). Mahat et al. (15); Osunde et al. (16, 17); and Chukwuma et al. (6), 2022 reported that incidence of alveolar osteitis was similar between suture-less and sutured groups, with rates typically ranging from 0% to 8%–10%. Sen et al. (1) and Mahat et al. (15) reported that soft-tissue healing and overall wound-closure outcomes were comparable or slightly better in the suture-less group, with fewer cases of delayed healing in the early postoperative period. Alkadi et al. (8), Hashemi et al. (5); Gay-Escoda et al. (7) specifically evaluated lingual or inferior alveolar nerve function and reported no significant difference in the incidence of transient or persistent paraesthesia between suture-less and sutured techniques.

Overall, the findings of the present study favours sutureless technique but the evidence is limited due to heterogeneity and risk of bias. Apart from these variables, No significant differences exist in infection or bleeding incidence between secondary and primary closure in the included studies. Sutureless and multiple sutures techniques after third molar surgery show comparable complication rates, including infection, alveolar osteitis (dry socket), hemorrhage, and wound dehiscence. However, limited sample size, and heterogeneity across studies arising from flap types (e.g., envelope vs. triangular) and follow-up protocols, are major limitations that limit the generalisability of the findings.

Key limitations include small sample sizes in several included trials, differences in follow-up schedules, and inconsistent use of pain-scale metrics and time-points. Many studies used VAS or NRS inconsistently across days, which may have contributed to the high *I*^2^ values and attenuated effect-size estimates. Further large-scale, multicenter RCTs with standardized outcome measures (e.g., VAS-based pain, gonion–lateral canthus-measured swelling, and interincisal opening at fixed days 1, 3, 7, and 14) are needed to clarify the net effect of suture-less vs. multiple-suture closure.

## Conclusion

The current metaanalysis demonstrates that suture-less wound closure after surgical extraction of mandibular third molars yields comparable outcomes to multiple suture in terms of post-op pain, edema, and trismus. Although no statistically significant superiority was demonstrated for either approach, suture-less wound closure appears to be a safe and reasonable clinical alternative that may help to minimize postoperative pain, facial swelling, and trismus.

## Data Availability

The original contributions presented in the study are included in the article/Supplementary Material, further inquiries can be directed to the corresponding author.
